# New genus and species of calanoid copepods (Crustacea) belonging to the group of Bradfordian families collected from the hyperbenthic layers off Japan

**DOI:** 10.3897/zookeys.951.49990

**Published:** 2020-07-22

**Authors:** Sota Komeda, Susumu Ohtsuka

**Affiliations:** 1 Takehara Station, Setouchi Field Science Center, Graduate School of Integrated Sciences for Life, Hiroshima University, 5–8–1 Minato-machi, Takehara, Hiroshima, 725–0024, Japan Hiroshima University Hiroshima Japan

**Keywords:** Bradfordian families, Clausocalanoidea, Diaixidae, hyperbenthos, Tharybidae

## Abstract

A new genus and species of calanoid copepods belonging to the group of Bradfordian families, *Pogonura rugosa***gen. et sp. nov.**, is described from the deep-sea hyperbenthic layers off Nagannu Island, Okinawa Prefecture, southwestern Japan. *Pogonura***gen. nov.** is similar to another Bradfordian genus *Procenognatha* in sharing the following characteristics: (1) segmentation of the antennule, fused segments II–IV, X–XI, XXVII–XXVIII in females and II–IV, X–XII, XXVII–XXVIII, right XXII–XXIII in males; (2) retained setae on the ancestral segments I–IV of the antennary exopod; (3) setules on the mandibular gnathobase; (4) 3 sclerotized setae on the maxillary endopod; (5) absence of sensory seta on the maxilliped; (6) large spinules on the posterior surface of the rami of legs 2 and 3; and (7) setation and segmentation of female leg 5. *Pogonura***gen. nov.** is distinctly distinguished from *Procenognatha* by the following features: (1) reduction of a seta on the ancestral segment IX of the antennary exopod, (2) 8 setae (7 in *Procenognatha*) on the maxillular exopod, (3) 5 brush-like setae (6 in *Procenognatha*) on the maxillary endopod, and (4) reduction of right endopod of male leg 5. The systematic position of *Pogonura***gen. nov.** in the Bradfordian families is also discussed. Although this new genus shares synapomorphies with some diaixid genera, an assignment of this genus to any Bradfordian family should be pending until the taxonomy of this family group is clearly settled.

## Introduction

Some clausocalanoidean families of calanoid copepods are characterized by the presence of chemosensory setae on the maxillary endopods, and in some taxa, on the maxillules and maxillipeds (Bradford 1973; Nishida and Ohtsuka 1997) and are called the Bradfordian families (Ferrari and Steinberg 1993). These groups are distributed in various marine habitats including both the pelagic realm and the hyperbenthic layers of the oceans (Bradford-Grieve 2004). Recently, many new families and genera collected from deep-sea hyperbenthic layers were established in the Bradfordian group (Ferrari and Markhaseva 1996; Ohtsuka et al. 2002, 2003; Markhaseva et al. 2008; Markhaseva and Schulz 2009). So far, seven families of the Bradfordian group have been recognized: Diaixidae Sars, 1902; Kyphocalanidae Markhaseva & Schulz, 2009, Parkiidae Ferrari & Markhaseva, 1996; Phaennidae Sars, 1902; Rostrocalanidae Markhaseva, Schulz & Martinez Arbizu, 2008; Scolecitrichidae Giesbrecht, 1892; and Tharybidae Sars, 1902.

According to Bradford (1973), the Scolecitrichidae and Phaennidae are families that are well defined by the armature of sensory elements on the maxillae, whereas the Diaixidae and Tharybidae are not as clearly diagnosed. Markhaseva et al. (2014) considered only *Tharybis* as a member of Tharybidae based on an autapomorphic character, an enlarged and vaulted arthrite of the maxillulary praecoxa which can differentiate between Tharybidae and Diaixidae. *Undinella* and *Brodskius* were conventionally included in Tharybidae (e.g., Markhaseva et al. 2014). Recently, analyses of relationships among the Bradfordian genera were performed by Markhaseva and Ferrari (2005) and Laakmann et al. (2019). Laakmann et al. (2019) also conducted a molecular-based phylogenetic analysis of these families but concluded that relationships between these seven families or 15 genera were not supported, except for the closeness between *Procenognatha* (conventionally assigned to Diaixidae) and *Tharybis* (Tharybidae).

The present paper deals with a description of a new genus and species of calanoid copepods belonging to the Bradfordian family collected from the deep-sea hyperbenthic layers off Nagannu Island, Okinawa Prefecture, southwestern Japan. The systematic position of this new genus is also discussed.

## Materials and methods

Copepods were collected from the deep-sea hyperbenthic layer off Nagannu Island, west of Okinawa Prefecture, southwestern Japan (26°19.23'N, 127°26.35'E, depths of 595–627 m) on May 21, 2011, using a sledge net (mouth area of 1450 × 326 mm, mesh size of 0.33 mm; see Ohtsuka et al.1992) towed along the sea bottom for 30 minutes at 2 knots. Samples were fixed with 10% neutralized formalin seawater immediately after capture. Type specimens are deposited at the National Museum of Nature and Science, Tsukuba, Japan (NMST-Cr 27413–27415). The morphological terminology follows Huys and Boxshall (1991).

## Taxonomy

**Order Calanoida Sars, 1901**

**Superfamily Clausocalanoidea Giesbrecht, 1893**

### Genus Pogonura gen. nov.

Taxon classificationAnimaliaCalanoidaClausocalanoidea

16409F26-3072-5FBF-8562-D5B256B5955E

http://zoobank.org/FC4F858B-9AC7-4D82-B5A7-FD861A8DF4A1

#### Diagnosis.

**Female.** Body compact. Cephalosome incompletely fused to first pediger with suture line dorsally and laterally visible. Fourth and fifth pedigers completely fused, weakly produced posteriorly into round lobes. Rostrum produced ventrally, with pair of filaments. Genital double-somite symmetrical, with pair of seminal copulatory pores and seminal receptacles; seminal copulatory pores ovaliform; genital operculum ventrally located midway; two spiniform setae located ventrolaterally, as long as genital double-somite. Antennule 24-segmented, with ancestral segments II–IV, X–XI, and XXVII–XXVIII fused; II–IV, VII, X–XI, XIV, XVI, XXI and XXVII–XXVIII with aesthetasc. Setal formula of antennary exopod as follows: 1, 1-1-1, 1, 1-1, 1, 0, 3. Mandible with gnathobase having 1 triangular ventral tooth, 5 chitinized teeth, 16 long setules, and 1 dorsal seta. Maxillulary exopod with 8 setae. Maxillary endopod with 5 brush-like setae and 3 sclerotized setae. Maxilliped with syncoxa having 1, 2, 3, 3 sclerotized setae. Legs 1–4 of typical clausocalanoidean segmentation and setation. Posterior surface of legs 2 and 3 with an exopodal spinule and 3 endopodal spinules. Leg 5 uniramous, 2-segmented, distal segment with 3 lateral process and 1 articulated spine.

**Male.** Body similar to that of female. Fusion between cephalosome and first pediger and between fourth and fifth pedigers resembling those of female. Genital somite with gonopore on left side. Rostrum as in female. Right antennule 22-segmented, with ancestral segments II–IV, X–XII, XXII–XXIII, and XXVII–XXVIII fused. Left antennule 23-segmented, with ancestral segments II–IV, X–XII, and XXVII–XXVIII fused. Antenna, mandible, maxillule, maxilla, maxilliped and legs 1–4 similar to those of female. Leg 5 complex in structure. Right leg uniramous, endopod absent; expod 2-segmented. Left leg biramous with 1-segmented endopod; exopods 2-segmented, decorated by various armatures; distal part of exopod with rugose plate.

#### Remarks.

Because *Pogonura* gen. nov. has brush-like sensory setae on the maxillary endopod, it can be assigned to one of the Bradfordian families. The new genus can be tentatively included in Diaixidae because it fits the familial diagnosis proposed by Markhaseva et al. (2014), except for the proximal basal endite of the maxillule with 3 setae (vs. 4 setae typical for the Diaixidae) and 2-segmented exopods of both legs 5 of the male.

The present new genus also shares the following characteristics with the diaixid genus *Procenognatha* (Markhaseva and Schulz 2010): the maxilliped carries no specialized sensory setae; legs 2 and 3 carry 3 large spinules on the posterior surface; and leg 5 of the female is uniramous, 2-segmented, with the distal segment having 3 processes and 1 articulated spine.

The male of the present new species has complex structures on leg 5, which can be seen in other diaixid genera such as *Anawekia* and *Diaixis*. These three genera have rows of setules and/or spinules on the left exopod of leg 5 [cf. figs 7 and 9 in Othman and Greenwood (1994); figs 11 and 12 in Andronov (1979)], and these setulae and/or spinules seem to be homologues in position and shape. However, *Anawekia* and *Diaixis* have some derivative characteristics: (1) the posterior corner of the prosome, leg 4, and the urosome of both sexes are asymmetrical; (2) female leg 5 is totally reduced; and (3) the left endopod of male leg 5 is reduced.

According to Markhaseva et al. (2014), the family Diaixidae has hitherto accommodated 15 genera. *Pogonura* gen. nov. is differentiated from these diaixid genera by the following features (morphological data from Markhaseva et al. 2014): (1) the genital double-somite of the female has a symmetrical pair of long spiniform setae (only *Pogonura* gen. nov.), (2) the distal part of the left exopod on male leg 5 has a rugose plate (only *Pogonura* gen. nov.), (3) the ancestral segments XI–XII of the male antennule are fused (shared by *Pogonura* gen. nov., *Byrathis*, *Diaixis*, *Paraxantharus*, *Procenognatha* and *Xantharus*), (4) the setal formula of the antennary exopod is 1, 1-1-1, 1, 1-1, 1, 0, 3 (only *Pogonura* gen. nov.), (5) the mandibular gnathobase has long setules (*Pogonura* gen. nov., *Cenognatha*, *Neoscolecithrix*, *Paraxantharus* and *Procenognatha*), and (6) the maxillary endopod has 3 sclerotized setae (*Pogonura* gen. nov. and *Procenognatha*).

#### Etymology.

The new generic name is derived from two Greek words *pogon*, meaning “beard”, and *oura*, meaning “tail”, to denote the paired setae like moustache on the genital double-somite of the female. Gender feminine.

#### Type species.

*Pogonura rugosa* sp. nov. (original designation).

### Pogonura rugosa

Taxon classificationAnimaliaCalanoidaClausocalanoidea

gen. et sp. nov.

487A0E5C-5DC5-5D47-B64C-BC3AC74D1F13

http://zoobank.org/6C0D4F63-BDD0-41A7-88D3-8D7D2EC9BB3C

Figs 1 , 2 , 3 , 4 , 5 , 6 , 7 , 8

#### Material examined.

***Holotype.*** One ♀; whole body in vial (NSMT-Cr 27413). ***Allotype.*** One ♂, dissected and appendages mounted on glass slide, body in vial (NSMT-Cr 27414). ***Paratype*.** One ♀, dissected and appendages mounted on glass slide, body in vial (NSMT-Cr 27415). Body length. Adult female: 1.69 mm (holotype), 1.73 mm (paratype). Adult male: 1.71 mm (allotype).

#### Description of adult female.

**Figure 1.**
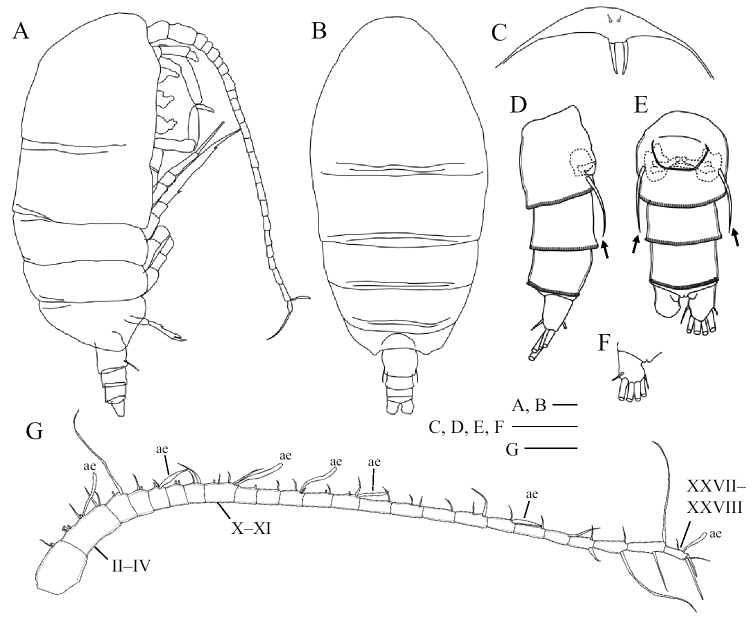
*Pogonura rugosa* gen. et sp. nov., adult female, paratype **A** lateral habitus **B** dorsal habitus **C** rostrum **D** urosome, lateral view **E** urosome, ventral view **F** caudal rami, dorsal view **G** right antennule; ae, aesthetascs. Arrows on **D** and **E** indicate twin spiniform setae on genital double-somite. Scale bars: 0.1 mm.

Body (Fig. 1A, B) weakly sclerotized; cephalosome incompletely fused to first pediger with future line dorsally and laterally visible; fourth and fifth pedigers completely fused; posterolateral corners of prosome extending posteriorly, rounded and covering one-third of genital double-somite. Rostrum (Fig. 1C) produced ventrally, with pair of frontal filaments distally. Urosome (Fig. 1D, E) 4-segmented; genital double-somite symmetrical with pair of seminal copulatory pores and seminal receptacles (Figs 1D, E, 8); seminal copulatory pores ovaliform; seminal receptacles narrow near the seminal copulatory pores and becoming semicircular in the inner part; genital operculum semicircular, ventrally located midway; two spiniform setae located ventrolaterally, as long as genital double-somite (Figs 1D, E, 8, indicated by arrows). Caudal rami (Fig. 1D–F) symmetrical, about 1.2 times as long as wide; seta I reduced, short seta II dorsally, seta III–VI long, short seta VII ventrally.

Antennule (Fig. 1F) 24-segmented, exceeding posterior border of third pediger; ancestral segments II–IV, X–XI and XXVII–XXVIII fused; armature as follows: I–2, II–3 (2+1ae?), III–2+1ae, IV–2, V–2, VI–2, VII–2+1ae, VIII–2, IX–2, X–2, XI–2+1ae, XII–1, XIII–1, XIV–2+1ae, XV–2, XVI–2+1ae, XVII–1, XVIII–2, XIX–1, XX–2, XXI–1+1ae, XXII–1, XXIII–1, XXIV–1+1, XXV–1+1, XXVI–1+1, XXVII–2, and XXVIII–2+1ae.

**Figure 2.**
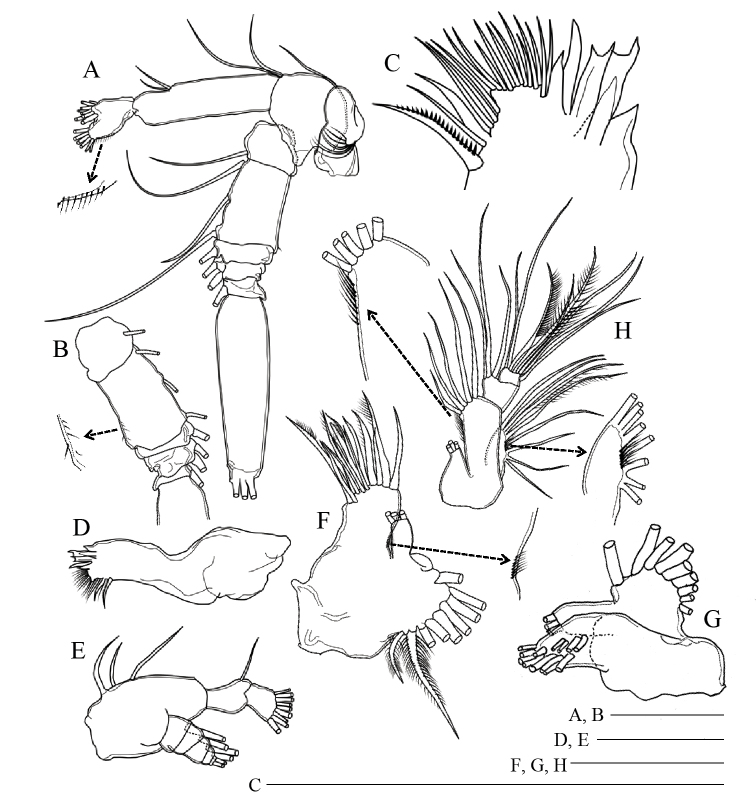
*Pogonura rugosa* gen. et sp. nov., adult female, paratype **A** right antenna **B** exopod of right antenna, other side **C** gnathobase on right mandible **D, E** right mandible **F, G** praecoxa and coxa of left maxillule **H** basis, endopod, and exopod of left maxillule. Scale bars: 0.1 mm.

Antenna (Fig. 2A, B) with 1 seta and row of long setules on coxa; basis with 2 setae at inner distal corner; exopod 7-segmented; ancestral segments II–IV fused and VI–VII incompletely fused without suture line, setal formula of 1, 1-1-1, 1, 1-1, 1, 0, 3; fused segments II–IV having row of fine setules along outer distal margin; endopod 2-segmented, proximal segment with 2 setae, distal segment bilobed, bearing 8 setae on inner lobe and 6 setae and short setules on outer lobe.

Mandible (Fig. 2C–E) having gnathobase with triangular ventralmost tooth, 5 chitinized teeth, 16 long setules and dorsal seta; palp with basis having 3 inner setae; endopod 2-segmented; proximal endopodal segment bearing 1 seta, distal segment with 9 setae; exopod 5-segmented, with setal formula of 1, 1, 1, 1, 2.

Maxillule (Fig. 2F–H) with 9 terminal and 4 posterior setae on praecoxal arthrite; coxal endite with 3 setae; coxal epipodite with 9 setae; proximal and distal basal endites having 3 and 5 setae, respectively; proximal and distal segments of endopod with 3 and 8 setae, respectively; exopod with 8 setae; rows of setules on arthrite, basis and exopod.

**Figure 3.**
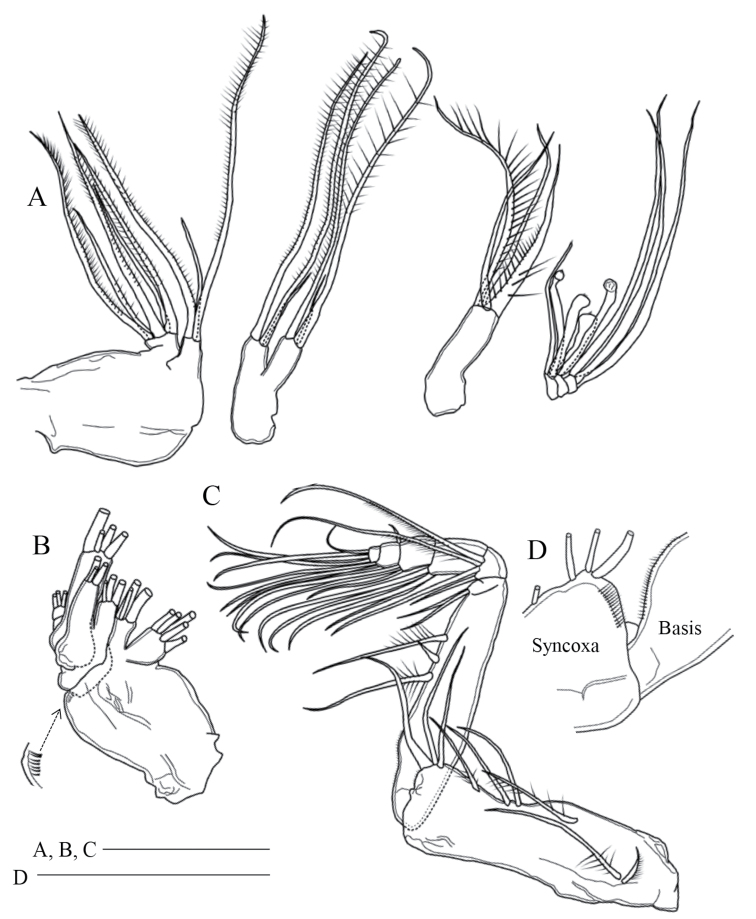
*Pogonura rugosa* gen. et sp. nov., adult female, paratype **A** right maxilla **B** right maxilla, other side **C** right maxilliped **D** rows of setulae on right maxilliped. Scale bars: 0.1 mm.

Maxilla (Fig. 3A, B) with 2 praecoxal and 2 coxal endites having 5, 3, 3, and 3 setae, respectively; basis with 1 well-chitinized and 3 slender setae; endopod 3-segmented, with 3 sclerotized and 5 brush-like setae of various length, proximal segment with 3 brush-like setae (1 slender, 1 short and stout, 1 moderate), middle segment with 2 brush-like setae (1 short and stout, 1 moderate), distal segment with 3 sclerotized setae.

Maxilliped (Fig. 3C, D) with syncoxal endites having 1, 2, 3 and 3 setae; row of fine setules at syncoxal distal corner and along basal inner margin; basis with 3 setae midway; first endopodal segment almost incorporated into basis; first to sixth endopodal segments with 2, 4, 4, 3, 3+1, and 4 setae, respectively.

**Figure 4.**
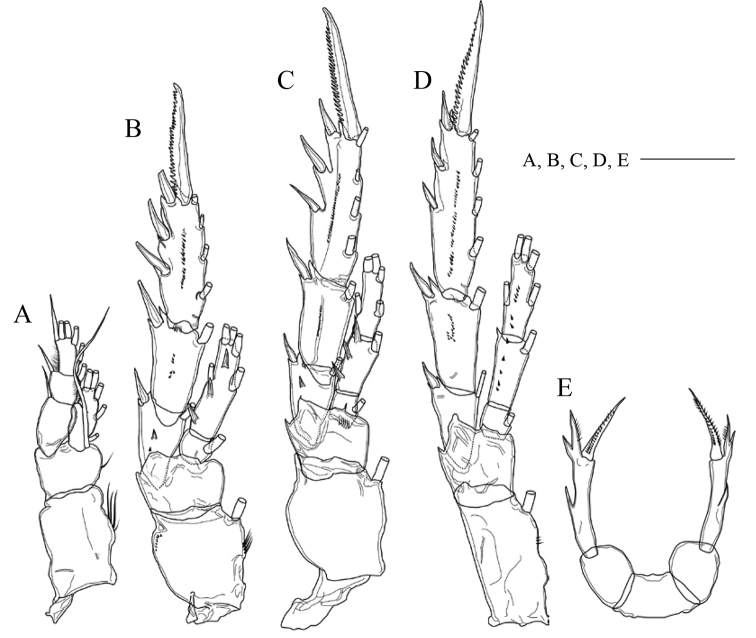
*Pogonura rugosa* gen. et sp. nov., adult female, paratype **A** leg 1, anterior side **B** leg 2, posterior side **C** leg 3, posterior side **D** leg 4, posterior side **E** leg 5. Scale bar: 0.1 mm.

Seta and spine formulae of legs 1–4 are shown in Table 1. Leg 1 (Fig. 4A) with medial long setules on coxa and basis; von Vaupel Klein organ (Vaupel Klein 1972) distinct on anterior surface of endopod; distal seta of basis twice as long as endopod; distal segment of exopod with outer row of setules. Leg 2 (Fig. 4B) with coxa having row of fine setules midway and row of fine spinules at distal outer corner; first exopodal segment with 1 large and 1 minute prominence on posterior surface; distal endopodal segment having 3 large prominences on posterior surface. Leg 3 (Fig. 4C) with basis having row of minute setules at base of endopod on posterior surface; second endopodal segment having 3 large prominences on posterior surface. Leg 4 (Fig. 4D) with first and second segments of both rami having small prominences on posterior surface.

**Table 1.** Setal formula of legs 1–4 of *Pogonura rugosa* gen. et sp. nov. Roman numeral: spine, Arabic numeral: seta.

	Coxa	Basis	Exopod			Endopod		
			1	2	3	1	2	3
Leg 1	0-0	0-1	I-0;	I-1;	I, 1 ,3	0, 2, 3		
Leg 2	0-1	0-0	I-1;	I-1;	III, I ,4	0-1;	1, 2, 2	
Leg 3	0-1	0-0	I-1;	I-1;	III, I, 4	0-1;	0-1;	1, 2, 2
Leg 4	0-1	0-0	I-1;	I-1;	III, I, 4	0-1;	0-1;	1, 2, 2

Leg 5 (Fig. 4E) uniramous; coxae and intercoxal sclerite fused to form common base; basis broad, about 1.3 times as long as wide; exopod 1-segmented, ca. 4.4 times as long as wide, with 3 lateral processes and 1 terminal bipinnate spine.

#### Description of adult male.

**Figure 5.**
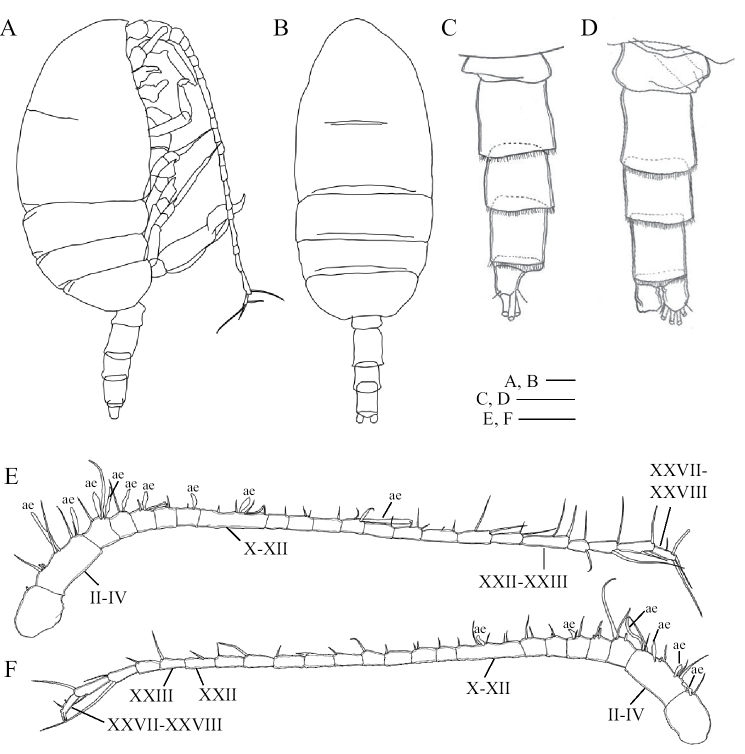
*Pogonura rugosa* gen. et sp. nov., adult male, allotype **A** lateral habitus **B** dorsal habitus **C** urosome, lateral view, left side **D** urosome, ventral view **E** right antennule **F** left antennule; ae, aesthetascs. Scale bars: 0.1 mm.

Body (Fig. 5A, B) weakly sclerotized like the female; fusion between cephalosome and first pediger and between fourth and fifth pedigers similar to those of female; posterolateral corners of prosome rounded, not extending posteriorly. Rostrum similar to that of female. Urosome (Fig. 5C, D) 5-segmented; gonopore located on the left side; small plate covering around gonopore; caudal rami similar to those of female.

Antennule asymmetrical in fusion patterns. Right antennule (Fig. 5E) 22-segmented; ancestral segments II–IV, X–XII, XXII–XXIII and XXVII–XXVIII fused; armature elements as follows: I–1, II–3 (2+1ae?), III–1+1ae, IV–2+1ae,V–2+2ae, VI–1+ae, VII–2+1ae, VIII–2, IX–1+1ae, X–1, XI–2+1ae, XII–2, XIII–1, XIV–2, XV–1, XVI–2+1ae, XVII–1, XVIII–3, XIX–2, XX–2, XXI–2, XXII–1, XXIII–1, XXIV–1+1, XXV–1+1, XXVI–1+1, and XXVII–1, and XXVIII–2+1ae. Left antennule (Fig. 5F): 23-segmented; ancestral segments II-IV, X-XII and XXVII-XXVIII fused; armature elements as follows: I–1+1ae, II–2+1ae, III–2+ae, IV–2+1ae, V–3, VI–3, VII–2+1ae, VIII–2, IX–2, X–2, XI–2+1ae, XII–1, XIII–1, XIV–2, XV–1, XVI–1, XVII–1, XVIII–1, XIX–1, XX–1, XXI–1, XXII–1, XXIII–1, XXIV–1, XXV–1+1, XXVI–1+1, XXVII–2, and XXVIII–2.

Other appendages similar to those of the female, except leg 5.

**Figure 6.**
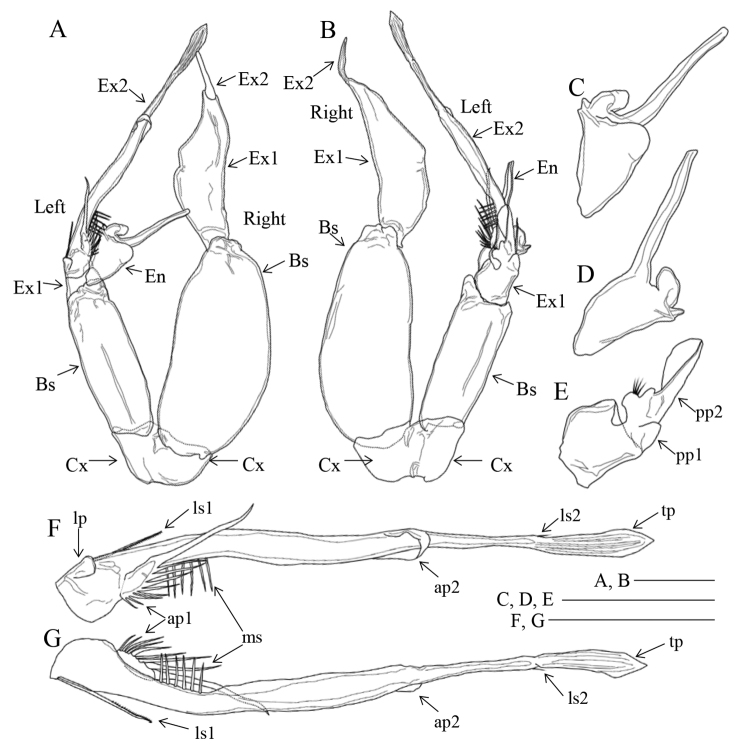
*Pogonura rugosa* gen. et sp. nov., leg 5 of adult male, allotype **A** anterior side **B** posterior side **C** left endopod, anterior side **D** left endopod, posterior side **E** proximal segment of left exopod, posterior side **F** distal segment of left exopod, anterior side **G** distal segment of left exopod, posterior side. Bs: basis, En: endopod, Ex1: proximal segment of exopod, Ex2: distal segment of exopod, pp1: proximal posterior plate, pp2: distal posterior plate, lp: lateral plate, ls1: proximal lateral spinule, ls2: distal lateral spinule, ap1: proximal anterior plate, ap2: distal anterior plate, ms: medial spinules, tp: terminal plate. Scale bars: 0.1 mm.

**Figure 7.**
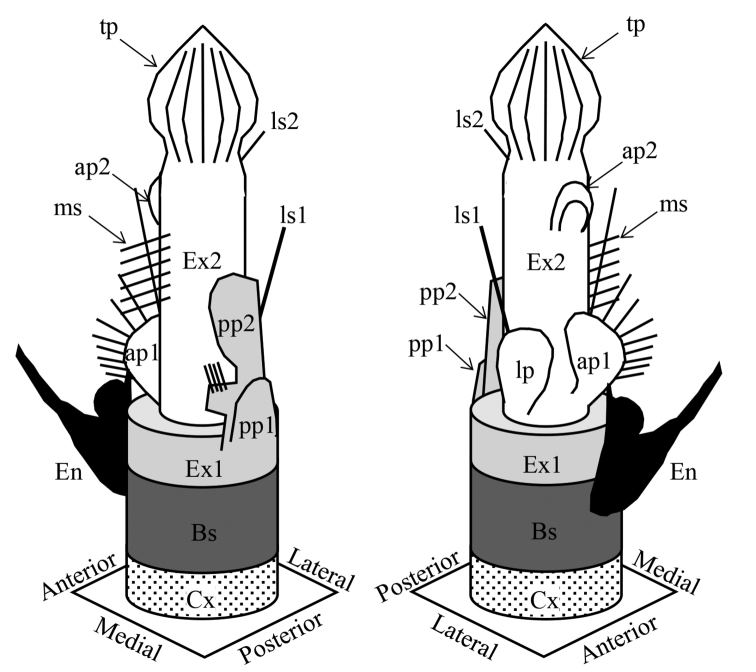
Schematic diagram of armatures on left leg 5 of male *Pogonura rugosa* gen. et sp. nov. Cx: coxa (with dots), Bs: basis (dark gray), En: endopod (black), Ex1: proximal segment of exopod (light gray), Ex2: distal segment of exopod (white), pp1: proximal posterior plate, pp2: distal posterior plate, lp: lateral plate, ls1: proximal lateral spinule, ls2: distal lateral spinule, ap1: proximal anterior plate, ap2: distal anterior plate, ms: medial spinules, tp: terminal plate.

Right leg 5 (Fig. 6A, B) uniramous; endopod absent; coxa small; basis robust, 2.2 times as long as wide; exopod 2-segmented, proximal segment plate-like, distal segment spiniform. Left leg 5 (Figs 6A–G, 7) biramous; coxa small; basis smaller than right basis and slender, 2.5 times as long as wide; endopod 1-segmented, plate-like, and having baculiform plate and semicircular plate; exopod 2-segmented and highly complex in structure; proximal segment of exopod having proximal plate without armament (“pp1” in Figs 6, 7) and distal plate, larger, semicircular plate with 4 setules (“pp2” in Figs 6, 7); distal segment of exopod elongate, 9.0 times as long as proximal segment; proximal part of distal segment having 6 medial spinules (“ms” in Figs 6, 7), lateral spinule (“ls1” in Figs 6, 7) and anterior plate with 9 spinules (“ap1” in Figs 6, 7), midpoint of segment having thin, curved plate (“ap2” in Figs 6, 7) anteriorly, and distal part having spinule (“ls2” in Figs 6, 7) and thin plate with crest (“tp” in Figs 6, 7).

#### Remarks.

**Figure 8.**
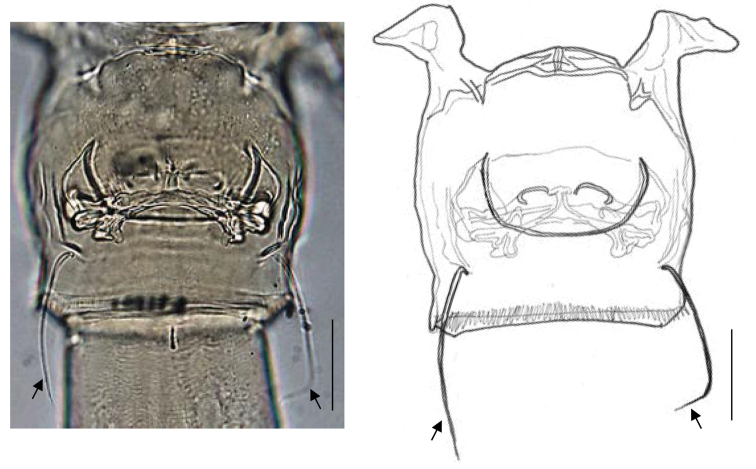
Ventral views of genital double-somite of female *Pogonura rugosa* gen. et sp. nov., paratype. Arrows indicate twin spiniform setae. Scale bar: 50 μm.

*Pogonura rugosa* gen. et sp. nov. has a symmetrical pair of spiniform setae on the genital double-somite of the female (Figs 1E, 8). *Diaixis centrura* Connell, 1981, *D. gambiensis* Andronov, 1979, and *D. trunovi* Andronov, 1979 also have armatures on the counterparts [figs 39 and 94 in Andronov (1979); fig. 4 in Connell (1981)]; however, those of *Diaixis* are asymmetrical and consist of fine spinules.

#### Etymology.

The specific name of the new species is derived from a Latin word *rugosa*, meaning “rugose”, to denote leg 5 of the adult male with many foliaceous armatures.

## Discussion

The females of *Pogonura rugosa* gen. et sp. nov. have a ventrolateral pair of spiniform setae on their genital double-somite (Figs 1, 8). These armatures are similar to leg 6 possessed by females of podoplean copepods in the position (symmetrical armatures on the ventrolateral of the genital double-somite) (cf. Huys and Boxshall 1991). However, these spiniform setae do not seem to be a homologue of leg 6. Generally, leg 6 comprises a symmetrical pair of basal processes with a few elements at the tip (at most 3 setae or spines on each process) and is connected to the inner muscles at the base (Boxshall 1982; Huys and Boxshall 1991). However, the spiniform setae of *P. rugosa* gen. et sp. nov. lack basal processes and are not connected with inner muscles like the podoplean’s leg 6. Some groups of calanoids generally have the genital operculum on their genital double-somite of adult females, which is considered a homologue of leg 6 (Bradford-Grieve et al. 2010).

*Pogonura* gen. nov. has 3 setae on the terminal exopodal segment of the maxilla, although almost all copepods have at most 2 setae on this segment (Ferrari and Ivanenko 2008). In the Clausocalanoidea, however, some genera of the family Aetideidae have 3 setae on their counterparts (cf. *Pseudeuchaeta vulgaris* Markhaseva, Mohrbeck & Renz, 2017; *Paracomantenna profunda* Markhaseva & Renz, 2019). This retention in setae can be considered as an ancestral state in the clausocalanoideans.

Markhaseva and Ferrari (2005) and Laakmann et al. (2019) attempted to morphologically classify the Bradfordian genera into three main groups by considering the setations of the maxillary endopods, the antennary exopods and the maxillipedal praecoxal endites [=syncoxal endites sensu Huys and Boxshall (1991)], viz., Group A (Diaixidae and Tharybidae), Group B (Phaennidae and Parkiidae), and Group C (Scolecitrichidae). Laakmann et al. (2019) simultaneously conducted a molecular phylogenetic analysis of the Bradfordian genera but failed to assign different families or genera into any robust group except for *Procenognatha* and *Tharybis*. *Pogonura* gen. nov. shares some plesiomorphies with Group A sensu Markhaseva and Ferrari (2005) in the setation of the maxillipedal syncoxa. In addition, members of Group A have the following other plesiomorphies: all setae on the ancestral segments I–IV of the antennary exopod are retained and no specialized chemosensory seta is observed on the maxillipedal syncoxa. As mentioned in Remarks, *Pogonura* gen. nov. shares the following synapomorphies with some diaxid genera: with *Procenognatha*, posterior spinules are present on legs 2 and 3 (Markhaseva and Schulz 2010) and with *Anawekia* and *Diaixis*, a row of spinules is found on the left exopod of male leg 5 (Andronov 1979; Connell 1981; Othman and Greenwood 1994). These synapomorphies of *Pogonura* gen. nov. imply their close relationships with these diaixids. According to Laakmann et al.’s (2019) molecular analysis, *Procenognatha* comprises a robust clade with *Tharybis* (Tharybidae). In the present study, an assignment of *Pogonura* gen. nov. to any Bradfordian family should be pending until the taxonomy of this family group is clearly settled.

## Supplementary Material

XML Treatment for Pogonura

XML Treatment for Pogonura rugosa
